# AGUIA: autonomous graphical user interface assembly for clinical trials semantic data services

**DOI:** 10.1186/1472-6947-10-65

**Published:** 2010-10-26

**Authors:** Miria C Correa, Helena F Deus, Ana T Vasconcelos, Yuki Hayashi, Jaffer A Ajani, Srikrishna V Patnana, Jonas S Almeida

**Affiliations:** 1 Department of Bioinformatics and Computational Biology, The University of Texas MD Anderson Cancer Center, 1515 Holcombe Blvd, Houston, TX 77030, USA; 2 Bioinformatics Laboratory, Laboratório Nacional de Computação Científica, Av Getúlio Vargas, 333 Petrópolis, Rio de Janeiro, Brazil; 3 Institute of Chemical and Biological Technology, Universidade Nova de Lisboa, Oeiras, Portugal; 4 Instituto Nacional de Metrologia, INMETRO, Av. Nossa Senhora das Graças 50 -prédio 6, 25250-020, Xerém - Duque de Caxias, Rio de Janeiro - Brazil; 5 Department of Gastrointestinal Medical Oncology, The University of Texas MD Anderson Cancer Center, 1515 Holcombe Blvd, Houston, TX 77030, USA; 6 The University of Texas MD Anderson Cancer Center, 1515 Holcombe Blvd, Houston, TX 77030, USA; 7 The University of Texas Medical School, 6431 Fannin St, Houston, TX 77030, USA

## Abstract

**Background:**

AGUIA is a front-end web application originally developed to manage clinical, demographic and biomolecular patient data collected during clinical trials at MD Anderson Cancer Center. The diversity of methods involved in patient screening and sample processing generates a variety of data types that require a resource-oriented architecture to capture the associations between the heterogeneous data elements. AGUIA uses a semantic web formalism, resource description framework (RDF), and a bottom-up design of knowledge bases that employ the S3DB tool as the starting point for the client's interface assembly.

**Methods:**

The data web service, S3DB, meets the necessary requirements of generating the RDF and of explicitly distinguishing the description of the domain from its instantiation, while allowing for continuous editing of both. Furthermore, it uses an HTTP-REST protocol, has a SPARQL endpoint, and has open source availability in the public domain, which facilitates the development and dissemination of this application. However, S3DB alone does not address the issue of representing content in a form that makes sense for domain experts.

**Results:**

We identified an autonomous set of descriptors, the GBox, that provides user and domain specifications for the graphical user interface. This was achieved by identifying a formalism that makes use of an RDF schema to enable the automatic assembly of graphical user interfaces in a meaningful manner while using only resources native to the client web browser (JavaScript interpreter, document object model). We defined a generalized RDF model such that changes in the graphic descriptors are automatically and immediately (locally) reflected into the configuration of the client's interface application.

**Conclusions:**

The design patterns identified for the GBox benefit from and reflect the specific requirements of interacting with data generated by clinical trials, and they contain clues for a general purpose solution to the challenge of having interfaces automatically assembled for multiple and volatile views of a domain. By coding AGUIA in JavaScript, for which all browsers include a native interpreter, a solution was found that assembles interfaces that are meaningful to the particular user, and which are also ubiquitous and lightweight, allowing the computational load to be carried by the client's machine.

## Background

The heterogeneity of data produced by biomedical research creates a serious challenge to the interoperability and consistent aggregation of data [1], which renders the development and maintainance of web applications correspondingly more time consuming and resource intensive [2]. This reinforces a preference for front-end applications that are automated and web-based as much as possible. The semantic web resource description framework (RDF) offers particular advantages in this regard, as its data structure can contain a combination of domain ontology components as well as the graphic rules ontology. The domain ontology predates the work described here in the sense that it was previously identified and is regularly and independently changed by the domain experts. The regular changes in the domain ontology typically reflect new data sources, but may also correspond to a novel understanding of an old relationship between data elements. We proposed such an ontology incubation process in 2006 [3] and a formal model in 2010 [4]; an example of its application to lung cancer research illustrates the use of the simple sloppy semantic database (S3DB) tool to identify and maintain the correspondingly fluid RDF stores [5]. By contrast, the AGUIA ontology described in this report is fixed: it is designed to mediate the automated presentation of the fluid domain ontology and is therefore independent from the domain ontology. The instantiation of the AGUIA ontology involves the specification of the links between the graphical user interface (GUI) ontology and the domain ontology. Where this association is not specified, AGUIA will use default values to produce a default and a rather dull interface that will respond to the topology of the domain ontology. The dependency between S3DB collections and items (tabs), and rules and statements (rows) for the domain ontology is graphically captured as tabbed navigation. Therefore, the context for the automated interface assembly work described in this report is that of accounting for user bias/context in the representation of a knowledge base. In other words, the domain ontology serves as an underlying structure to describe the data content of the web application that describes the concepts and their relationships in the specified domain. This ontology was created to store the data from clinical trials for gastrointestinal cancer. The graphic model is based on graphic rules that describe the graphic structure of the web application. This model contains the graphic components of the web application and their association with the domain ontology. The web application is able to assemble a GUI through this RDF data structure. The supplementary material contains more details about the structure of data entry and the construction/instantiation of each component of the model. It includes a video demonstrating how the same AGUIA application is alternatively pointed to different S3DB deployments (see Materials).

The web application AGUIA was developed to manage clinical data through clinical trials semantic data services. Semantic data services are data objects with domain-specific semantics and technology standards that are used to provide secure real-time access to existing data sources. In addition to providing the context for sharing information based on program needs, semantic data services also support the dispersed data ownership requirement that generally exists for these programs. In a nutshell, semantic data services allow for the discovery and management of semantic relationships across information systems in a timely manner and on a large scale [6]. In this context, the clinical trials semantic data services (CTSDS) developed within S3DB deployments are semantic data services (and include a SPARQL endpoint) directed toward clinical trials. Through the CTSDS, it is possible to capture common characteristics of a particular group of patients and generate results that can best be examined by the physician. For example, it is possible to capture the data for all patients who smoke and have the same tumor type, such as a grade 2, moderately differentiated tumor.

The goal of developing a specification and standard that autonomously assembles a graphical user interface for a data source has a tradition in web technologies that can be traced back as far as hypertext in the use of HTML elements to manipulate the web browser's document object model.

The emergence of semantic web technologies has not entirely overlooked the assembly of user interfaces. This is particularly clear in RDF elements such as rdfs:label or rdfs:comment [7]. These elements anticipate the need for interface descriptors by other semantic models. Upon close scrutiny of the specifications, one finds that such elements are indeed widely used. For example, in W3C's simple knowledge organization systems (SKOS), the definition of the relationship skos:broader [8] comes with the indication that its rdfs:label is "has broader." SKOS [9] is a simple RDF schema for knowledge organization systems (KOS) such as thesauri, classification schemes, subject heading systems and taxonomies within the framework of the semantic web. Like many other RDFS-Plus [10] modeling efforts, encoding this information in RDF is ultimately geared toward the facilitation of interoperability between computer applications by creating a data infrastructure ecosystem in which the semantics of data generation and usage are both explicit in the representation [11].

The initial motivation for the work described here was the development of a web application to manage clinical data within the Gastrointestinal Medical Oncology Department of MD Anderson Cancer Center. It was clear from the beginning of the project that the diversity of users/usages and the fluidity of the underlying data model were not compatible with a conventional web application with a static layout. This is because the underlying schema are not only continuously undergoing significant changes, but those changes are not always the same for all involved. Therefore, it was decided that the ideal interface would be an autonomous browser-based web application that would respond to one or more independent descriptions of the data schema and graphic model in order for it to be used in multiple projects/representations. To enable the automatic assembly of the web application, a set of RDF descriptors was created by re-sorting the knowledge model schema [4] of an S3DB database [3]. The web application would then be able to use these descriptors together with the ontology that is the clinical data project, which is also stored on S3DB, to assembly itself. This rationale follows a proposal by other researchers that a knowledge base requires more than terminology and assertion components (TBox and ABox, respectively). Translated to correspond with a modern emphasis on semantic web formalisms, the RDF descriptors could also include a GBox component. Whereas ABox and TBox are terms coming from description logic [12], GBox was proposed by Motik [13] to describe how that information should be displayed.

In the work described here, instead of seeking to identify high-level graphic relationships to maximize reusability, we have taken the opposite route. First, a suite of graphical user interfaces was developed in response to specific requests from clinical researchers, and only then was an effort made to identify design patterns that could be captured as a specialized RDF protocol. The justification for this reverse approach is that starting from a graphic configuration that was already meaningful in relation to a specific domain will lead to a set of descriptors that are more easily recognized - and used - by those domain experts when they engage information management systems.

In spite of the domain-rooted approach followed at the beginning of this work, the clinical-trial-driven interfaces also represent a starting point for a more generic formalism-oriented approach that was originally proposed for intelligent information presentation systems (IIPS) [2]. That early work found a natural extension in the emergence of extended markup languages and subsequently in semantic web formalisms, leading to efforts to model web site interfaces such asWebML [14] and OntoWebber [15]. Furthermore, as noted by Lei *et al. *[2], those models could be enhanced by also considering key user interface issues such as page layouts and graphical user interfaces. AGUIA considers these interface issues, such that an interface generated by AGUIA should allow for multiple user-specific layouts.

## Methods

### Methods: browser-based application development

AGUIA was developed using dynamic HTML (DHTML) concepts and technologies to create dynamic and interactive web pages. DHTML is not a programming language, but rather is a set of programming techniques that combine HTML, JavaScript, HTML DOM and CSS. DHTML enables dynamic elements to be created inside the web page: fine-grained configurations such as text, page styles (font color, size and others), element positions, etc., can be changed dynamically after the page is loaded [16]. The application delivered with this report makes extensive use of the dhtmlgoodies library [17], which provides low level support for basic graphic features such as calendars, tabs, folder trees and others. The Google code management system [18] provides open source hosting of this application, which is publicly available at http://aguia.googlecode.com/hg/index.html. All code is made available in the corresponding parent directory structure, with the open source project management tools available at http://aguia.googlecode.com.

Testing and evaluation was performed by tracking usage and response times. Screencasts of typical usage were recorded and are provided with this report (see Results). Note that in the S3DB system (see Materials), data modeling is performed by the users themselves through the definition of S3DB rules.

### Materials: semantic database web service

The S3DB, which we used as our database web service, is an infrastructure for distributed data servicing that relies on semantic web concepts for the bottom-up management of heterogeneous domains [4]. It provides a bridge between a mass of structured data annotated by using personal ontologies and a globally referenceable semantic representation indexed to controlled vocabularies [4]. The S3DB web service exposes its API through a read/write REST protocol, S3QL. representational state transfer (REST) is a coordinated set of architectural constraints that attempts to minimize latency and network communication, while at the same time maximizing the independence and scalability of component implementations. This is achieved by placing constraints on connector semantics, in contrast to other styles that focus on component semantics. REST enables the caching and reuse of interactions, dynamic substitutability of components, and processing of actions by intermediaries in order to meet the needs of an Internet-scale distributed hypermedia system [19]. The S3DB database is also capable of producing its output in a variety of formats such as tabular text, XML and JSON, in addition to the generation of RDF in n3 or XML [20]. The open source application is made publicly available for a variety of operating systems. The RDF language provides a simple and flexible way of representing knowledge by breaking data structures down into dyadic predicates (triples) [21]. In other words, RDF is a general-purpose language for representing information in the web [7] and providing interoperability between otherwise incompatible domain models and formats. The RDF has an official query language created by W3C, the SPARQL. The S3DB is also able to receive SPARQL queries, which are then internally converted into S3QL [22], as recently illustrated for the cancer genome data [23].

Ultimately, the S3DB web service was developed to test the hypothesis that a user-editable schema and streamlined interoperability would facilitate the acquisition of biomedical data within the biomedical context and by the biomedical domain experts themselves [3].

The S3DB deployment, and by association its query language, S3QL, uses SQL to operate a regular relational database backbone. Most existing S3DB deployments use postgreSQL or mySQL. Other SQL databases that have been tested do not appear to pose a major limitation at that level. The performance is that of the supporting database, to which we add an overhead of migrating user permissions between entities of the S3DB data model. An online tool, available at http://s3db-operator.googlecode.com, illustrates the inner workings of this last component.

The research prototype of this database is mature enough that a few deployments have been adopted by MD Anderson Cancer Center and are subject to the same strict security audits of any other research tool dealing with sensitive data: see "Internal Services" at http://bioinformatics.mdanderson.org/. Also a number of external, public tools use it to service large datasets such as those produced by the cancer genome atlas (TCGA). For an example, see the DNA copy number browser at http://cnviewer.googlecode.com/. Currently, the scalability of this web service is that of the relational backbone - which is indeed more stringent than, for example, a map-reduction store.

Additional, non-essential I/O, server-side functionality was achieved by developing small applications coded in the PHP language. An example is a function to export the application data to excel spreadsheets, as most browsers don't offer native support for ActiveX technology. As noted in the previous section, all essential components of AGUIA are native to the web browser - that is, they are coded entirely in JavaScript.

## Results

### RDF protocol and list of graphic rules

**Figure 1:**
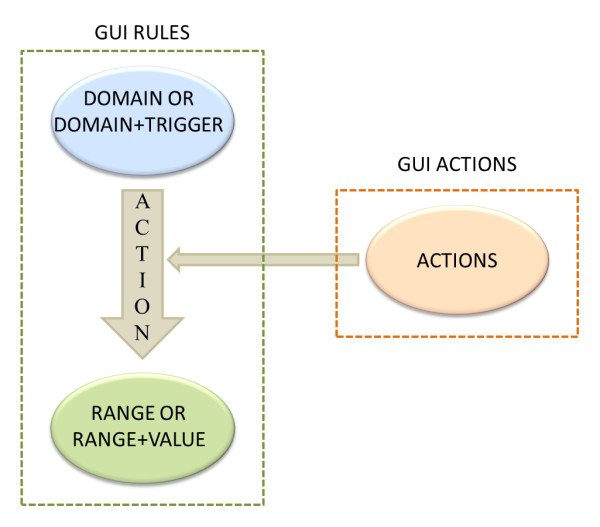
**Relationship between GUI rules and GUI actions**. Illustration of the relationship between GUI rules and GUI actions to generate RDF triples: GUI rules are S3DB rules predicated by S3DB items of the S3DB collection of AGUIA actions.

**Figure 2:**
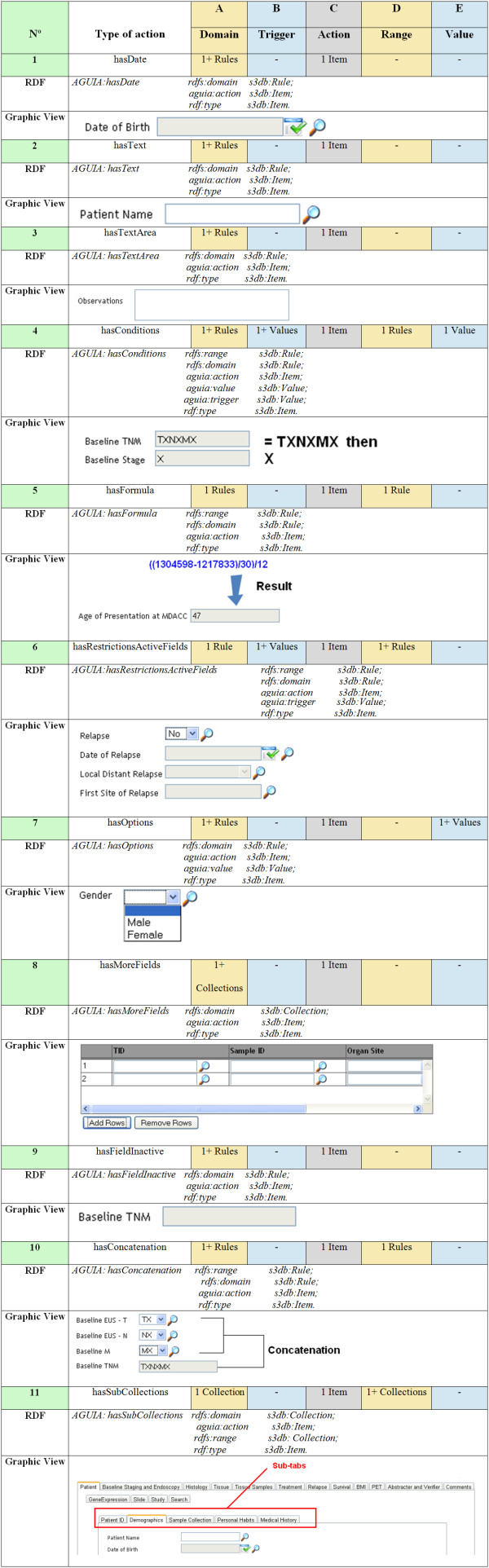
**Detail of the 11 × 5 relationships**. Details of the 11 × 5 relationships needed to configure automated assembly of the graphical user interface by the browser-based (pure JavaScript) AGUIA application. Each row corresponds to the 11 GUI actions, instantiated by S3DB items, tabulated against 5 GUI rules (columns), instantiated by S3DB rules. The 7th column is a description of the graphic component controlled by each of the 11 GUI actions. Note that S3DB is being used as a user-editable representation that is translated into standard RDF in this figure. The useful feature of S3DB as a tool, in addition to the user-editing tools, is that the distinction between domain and instantiation is always explicit [25].

The automatic assembly of the interface relies on two data sources, one being the target observational data and the other containing the graphic rules for the assembly of the interface. The graphic rules contain two parts: GUI actions and GUI rules (Figure 1), both of which are collections of an S3DB project. S3DB collections are described according to the RDF triple - *subject-predicate-object *- and S3DB rules are the subject or object of the RDF triple [4]. The GUI actions collection contains the actions that can be created. These are described in Figure 2. The GUI rules collection contains the instantiation of each graphic component. This collection contains the rules domain, range, action, trigger and value.

• Domain will contain the ID of the S3DB rule that will command the action.

• Range will contain the ID of the rule that will receive the action.

• Action will contain the action inserted in the GUI actions collection.

• Trigger will contain the value that will be tested.

• Value will contain the value that will be put in the range.

These collections compose the RDF triple. The GUI actions collection provides the predicate of the RDF triple for the GUI rules collection (see Figure 1). The GUI rules collection then instantiates each graphic component. The subject of the RDF triple can be a domain or a domain plus a trigger (the value that will be tested in the object if the predicate is some test action). The predicate corresponds to the action generated by the GUI actions collection and the object is composed of the range or the range plus a value (a value that will be placed on the range). The supplementary material includes examples that explain each of these rules and also provides the file for the project of the graphic rules (GUI rules project) with the GUI rules collection and GUI actions collection that contain data to download. They can be imported to S3DB just as in any other S3DB project.

Specifically, the web application requires the definition of up to 7 input parameters, three of which are mandatory - two are the locations for each of the two web services and the third is for an authentication token. The other four parameters will narrow the definition of the data elements on which the interface focuses and extend the range of data sources by allowing multiple deployments and users to be used. The access parameterization is extensively detailed and discussed in the documentation provided at the open source project hosting of AGUIA (see Methods).

**Figure 3:**
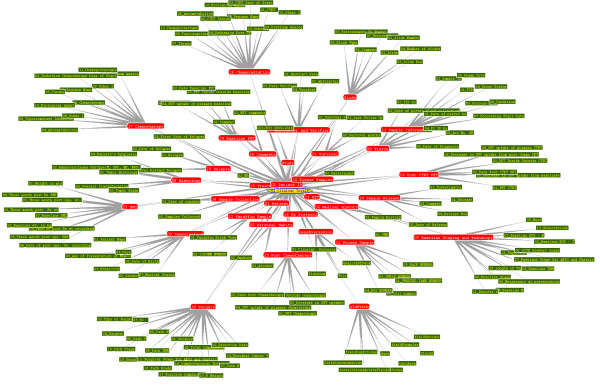
**Ontology of clinical trials data project**. User-defined ontology of the clinical trials data project in the Department of Gastrointestinal Medical Oncology, MD Anderson Cancer Center. The user describes the ontology by defining "rules," which in turn are instantiated by "statements." The oval node represents the root of the ontology; the red nodes represent rule elements that are collections of items; the green nodes represent rule objects that take literal values.

Both the data source and the graphic rules source are presently expected to be S3DB databases, accessed using a REST protocol, S3QL (see Materials). As discussed later, this reflects the current lack of standards for writing documents in the RDF more than it reflects a narrow focus of the S3DB prototype. The S3DB data acquisition effort described in this report was pursued in the Department of Gastrointestinal Medical Oncology at the University of Texas MD Anderson Cancer Center; hence, these data are of biomedical origin. Specifically, over a period of one year, clinical and biomedical researchers in this department submitted to an S3DB deployment a variety of clinical and biomolecular data totaling over 1 million independent S3DB "statements," which presently describe 1369 patients. These data were imported to S3DB through a script that receives the data in a tabular format. This script generates queries in S3QL, which transforms the data into S3DB RDF statements. Any S3DB project can be used to feed into the generic front-end AGUIA, although ideally AGUIA will also access a second S3DB project in which the AGUIA rules and actions are defined. The main operational advantage of relying on the S3DB web service is that the deletion, insertion and updating of data can be done through S3QL queries, whereas the reference SPARQL queries are limited to data retrieval. It is important to highlight that the data submission is completely decoupled from the configuration of the display. Consequently, it is easy to configure one or more alternative interfaces for the same data by targeting the RDF-based description of the domain (see Figure 3 for an example). More details about the data submission to S3DB can be found in the supplementary material. Figure 3 shows the ontology of the illustrative clinical trial project as a graph of user-submitted S3DB "rules" [5]. The report we reference includes graphic representations of the same RDF set using different RDF browsers that are both academic and commercial. Note in this figure that the patient ID collection plays a key role in aggregating the other collections. The supplementary material includes additional documentation and an extended discussion about how AGUIA handles the "main collection" and how the user works with this collection when performing a search, insertion or update of data.

**Figure 4:**
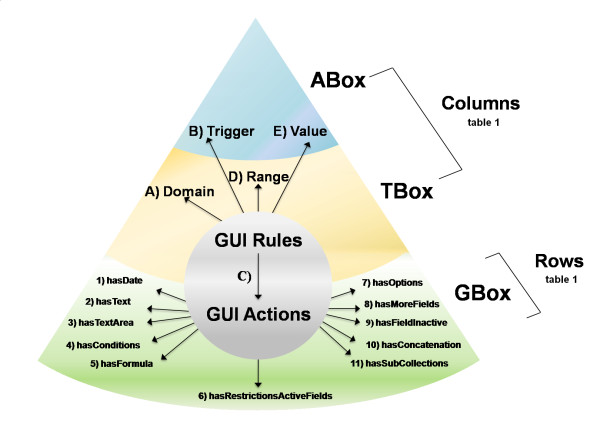
**Graphic model of S3DB items11 × 5 S3DB rules**. Graphic model as a set of 11 S3DB items (1-11) and a set of 5 S3DB rules (A-E). See Figure 2 for a translation into RDF schema by cross-tabulating the 5 GUI rules by the 11 GUI actions. The type of relationship for the GUI rules (S3DB items for the GUI actions -- GBox; S3DB rules and S3DB collections -- TBox; and S3DB values -- ABox) identifies the type of data that instantiate each of the 16 parameters of the model.

The AGUIA can be pointed to the URI of any of the red nodes in Figure 3 (S3DB collections) to start the process of assembling the automated graphical user interface. The graphic rules come from a second data source, which, unlike the first data source, comes from a project with a predefined ontology. More specifically, this second data source for the graphic clues is a distinct S3DB web service that instantiates a fixed set of S3DB rules (Figure 4). The RDF model proposed here was derived from those rules.

The graphic rules project (Figure 4 and Figure 2) is populated with statements that specify how to assemble the web application. In other words, by submitting statements that instantiate those rules, the user is configuring the graphical user interface as modular components. The graphic model described in Figure 4 corresponds exactly to the set of S3DB rules linking two object collections - GUI rules and GUI actions - with, respectively, 5 and 11 literal values. These rules (the 14 types of values) can be instantiated as many times as is required, and in any way needed to produce the desirable graphical appearance. This figure shows 3 parts of the knowledge base (KB): the GBox, TBox and ABox. The AGUIA GBox contains only 11 graphic components that can be used alone to compose the layout of a web application. Conventionally, the TBox indicates the terminological component, which in the AGUIA context has a domain and range that are S3DB rules, which themselves point to S3DB collections and/or S3DB rules. Finally, the ABox indicates the assertion component, which links to the context of AGUIA's GBox as values/literals that trigger AGUIA actions (Figure 4 and Figure 2).

As highlighted in the Introduction, the identification of this model was pursued by decomposing a diversity of layouts requested by clinical researchers such that any of the layouts requested can be automatically produced by AGUIA from instantiations of this model. The translation into the RDF schema of the relationships described by the 15 S3DB rules represented in Figure 4 is the essence of the modeling work detailed in Figure 2. In this figure each RDF row represents in RDF language the instantiation of each graphic component. For example, the date of birth is always a date, therefore the specification of the nature of the data can trigger its visualization. The supplementary material also provides examples of the instantiation of each graphic component in turtle language.

The goal of the work described here was to develop a front-end web application that may be reused for different projects with different needs. The use of the AGUIA web application was illustrated in two projects with widely varying needs: a project that contains clinical/molecular data collected from patients with gastrointestinal cancer, and a workshop project that contains data collected from participants in a workshop, including their home institution, research interests and personal data. The former test case was used to produce the illustrations in this report; the latter was used for illustration in the screencast video (see the supplementary material).

### JavaScript application

**Figure 5:**
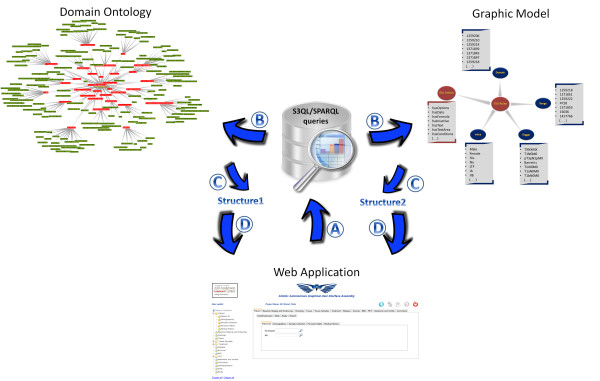
**Automated GUI assembly process**. Automated GUI assembly process: A) individual queries converted into independent URL calls in S3QL or SPARQL format; B) decomposition of the elements of the query that pertain to the domain description and to the graphic layout; C) translation of those query results into an AGUIA graphic document model (using notation 3: Project Collection Item; Rule Statement); D) merging of the two data structures back into the GUI object model, which is reflected back to the browser's own object model (DOM), as described in Figure 2.

The AGUIA web application is a JavaScript application developed to autonomously assemble the graphical user interface using the graphic annotation clues provided by the GUI rules and actions (Figure 4 and Figure 2). This means that any project based on S3DB can be automatically accessed through the graphical user interface assembled by this web application. The assembly of the GUI starts with queries in S3QL or SPARQL that are made to the S3DB web service. The result of these queries are grouped in only one data structure. This structure contains the association between the graphic components and the components of domain ontology, containing only the information necessary to assemble the web application (Figure 5). For the two case studies the observed response times of the S3DB system to each query were 0.4 seconds on average; the response times to assemble the full web application were under 11 seconds. Figure 5 depicts the functional architecture of AGUIA through which the flow of operations can be traced. Note that the S3DB deployments independently contain both the domain ontology and the graphic model. The domain ontology describes the data content in the web application (both TBox and ABox), whereas the graphic module describes the graphic structure of the web application. Recalling from Figures 1 and 4, the latter is divided into GUI actions and GUI rules. GUI actions contain the action that can be triggered by an assertion, for example, *hasDate *will trigger *create date field*. GUI rules are divided into a domain, range, trigger and value, as described in the section *RDF protocol and list of graphic rules. *Note also that this description of the architecture is further expanded in the supplementary material. That additional documentation includes information about response times and a video with a screencast of the real-time use of AGUIA to assess the performance/response times of queries and of the web application assembly.

Note that the absence of a graphical annotation (GBox) will not prevent the application from assembling the graphical interface automatically. The sole difference is that its structure will be based on only the structure of the relationships between the data elements. Therefore, the graphic annotations provided through the GUI rules and actions can also be thought of as a way to further direct the GUI assembly process. After it is assembled, the AGUIA is able to search, insert and update registres through S3QL and SPARQL queries that are made behind the scenes by GET HTTP calls. The supplementary material includes explanations and screencast illustrations of how to search, insert and update data using AGUIA.

**Figure 6:**
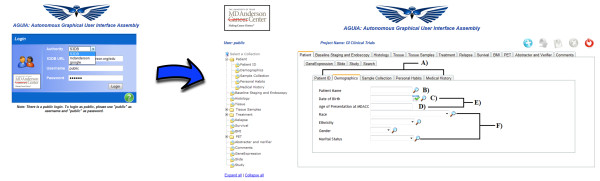
**Snapshot AGUIA**. Snapshot of the logon screen when AGUIA is pointed to GI clinical trials S3DB web service, followed by the start page assembled by AGUIA when configured to target resource "Patient ID." Selecting another resource, say "Tissue," will produce the exact same graphic interface as if the initial target had been that resource: the operation of AGUIA is fully based on representation state transfer (REST) calls. Part A shows sub-tabs; the action used to produce this field is "hasSubCollections." Part B shows a text field; the action used to produce this field is "hasText." Part C shows a date field; the action used to produce this field is "hasDate." Part D shows a formula field; the action used to produce this field is "hasFormula." Part E shows inactive fields; the action used to produce these fields is "hasFieldInactive." Part F shows options fields; the action used to produce these fields is "hasOptions."

The AGUIA web application is currently used by distinct groups of users in the Departments of Biostatistics, Bioinformatics and Gastrointestinal Medical Oncology at MD Anderson Cancer Center. Figure 6 depicts a snapshot of AGUIA when it is pointed to the gastric oncology clinical trial research database of the Gastrointestinal Medical Oncology Department. Both goals highlighted in the Background section for this application - the autonomous assembly of the user interface in the browser in response to the invocation of an independent GBox descriptor - were fully achieved. Consequently, the application was shown to accommodate the characteristically volatile schemas and to automatically assemble new interfaces in response to changes in the data model, without the need for additional coding. As noted in the Background, data schemas were observed to change to reflect both new research data and new researchers.

The user can test AGUIA to this database through a public login (url:http://ibl.mdanderson.org/edu username and password: public). This demonstration project describes fictitious patients but the data structure is exactly the same as that in use for gastric cancer clinical trials. The video in the supplementary material describes the login in this project and allows the viewer to see the assembly of the web application. By comparing Figure 6 with the ontology of the data store (Figure 3), it is apparent that a substantial number of GUI rules and GUI actions must be in use for this clinical data service. The list of S3DB rules (RDF describing the clinical domain) and the list of GUI actions and rules that modulates the assembly of the graphical user interface are provided with the supplementary material, as part of the documentation of AGUIA at the open source repository. This material includes a file in turtle/n3 format that provides the graphic rules and an exhaustive discussion of how each GUI action is interpreted by AGUIA. Examples of its use in the gastric oncology case study are also provided as supplementary material at the same URL.

The relevance of the assembly of the interfaces illustrated in Figure 6 in response to REST calls is that the navigation of the interface itself is a succession of REST calls. For example, the opening page was triggered by the following URL being sent to the web browser:

http://aguia.googlecode.com/hg/index.html?URLDATA=http://some_url/clinicaltrials|C1207734&KEYDATA=xxxxxxx&URLGUI=http://some_url/clinicaltrials|P1588674&KEYGUI=xxxxxxx

Starting with the domain URL, note that it targets the project hosting document directly. This illustrates an additional feature of JavaScript applications, which, being native to the web browser, remove the distinction between code hosting and application hosting - because they are the same. This parameterization of the call contains two locations and two authentications. For security reasons the actual URL and the access keys were replaced with "some_url" and "xxxxxx." The first location contains the URL of the data plus C1207734, which indicates the unique identifier of resource "PatientID." The second location contains the URL of the graphic rules plus P1588674, which indicates the instantiation of the GUI actions and rules used for the autonomous assembly. Note also that the authentication tokens, KeyDATA and KeyGUI, can be distinct, reflecting the possibility of different users seeing and configuring entirely distinct graphic interfaces for the same data.

### Testing/evaluation

The test/evaluation of the illustrative case study involved 1369 patients, which corresponded to approximately 1 million independent S3DB "statements" [4], as highlighted in the RDF protocol and list of graphic rules sub-section. The test/evaluation was divided into the following steps: application assembly, search, and view, insertion and update of data.

#### - Application assembly

The illustrative application described in this report is assembled in about 35 seconds if all graphic components are used, and in about 29 seconds if only one of them is invoked. In either case the data store has to be reached and the GBox retrieved and processed, which accounts for most of the assembly time. After the application has been completely assembled, all subsequent actions involving rearrangements of components of the interface become nearly instantaneous because all graphic components are contained in the memory and no further consultation of the store's GBox descriptors is needed. For all practical effects, the browser-based application is equivalent to a stand-alone application except for the important fact that no "download" and "installation" steps are needed.

#### - Search

The search operation of the application can be simple when it involves only one node (RDF resource) and complex when it involves more than one node. In either case the search operation consists of translating the use of graphic elements assembled as unstructured by the GBox into SPARQL queries, which are then issued back to the data store. For an example query and the screencast showing the graphic operations involved in generating them, see the supplementary material at [http://sites.google.com/site/aguiadocumentation/documentation/how-to-search]. Both the single node and multiple node scenarios are analyzed hereafter by considering three scenarios of increasing complexity.

First case: Search involving only one node (one level)

**Figure 7:**
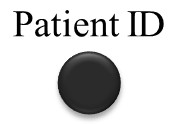
**One node search**. Search involving only one node.

In this case the search is realized directly (one level), for example, the search by participant number of all patients of the *Patient ID *collection (Figure 7).

Second case: Search involving one parent node and one child node (two levels)

**Figure 8:**
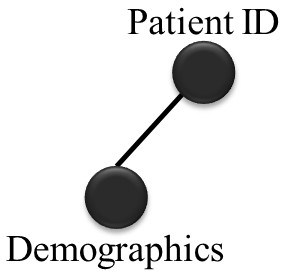
**Two nodes search**. Search involving one parent node and one child node.

In this case the search is performed at two stages (see Figure 8). In the first level the query contains the parent node (*PatientID*) and the second level contains only one child node (*Demographics*). An example of this type of search is a search by participant number and name of all the patients in the *Patient ID *and *Demographics *collections, respectively.

Third case: Search involving one parent node and two child nodes (two levels)

**Figure 9:**
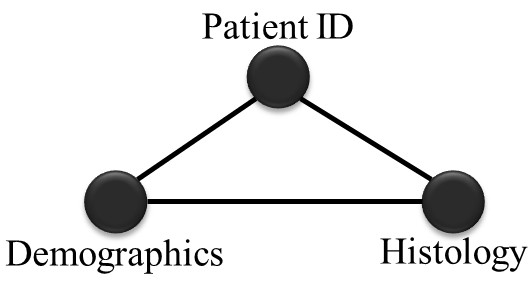
**Three nodes search**. Search involving one parent node and two child nodes.

In this case the search is again performed at two stages (see Figure 9). For example, a search by participant number, gender and tumor grade of all patients, seeking to identify those of *female *gender with a *G2 moderately differentiated *tumor, would involve the *Patient ID*, *Demographics *and *Histology *collections, respectively.

#### - Execution time

**Figure 10:**
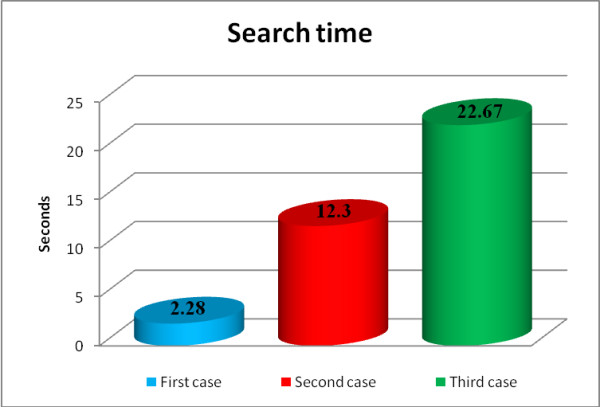
**Search time**. Search times for the three cases.

The three preceding scenarios were timed to assess the performance of the application. Figure 10 illustrates the length of time required to realize a search in each one of the cases.

The results depicted in Figure 10 show that each child node added 10 seconds to the search time. The analysis of the code execution shows that this corresponds to the addition of each node to the SPARQL query, suggesting linear scaling for the query performance.

#### - View, insertion and update

**Figure 11:**
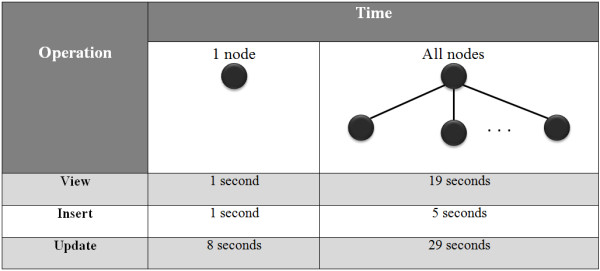
**Execution time to realize view, insert and update**. Time required to realize each one of the operations (view, insert and update), with one or all nodes.

The illustrative web application was configured to view, insert and update data one patient at a time. This takes place in one of two possible ways: View, insert and update patient data to only one node (only one collection on S3DB); or view, insert and update all patient data to all nodes (all collections on S3DB). Figure 11 describes these two scenarios, including the time associated with each operation in the context of the illustrative clinical trial application and a data store of 1 million statements. As reflected by those values, the process of data store indexing is the point at which semantic databases are comparatively slow.

The performance and operation of the prototype can be seen in a screencast video in the supplementary material (http://sites.google.com/site/aguiadocumentation/video). This video shows a typical usage of the AGUIA interface.

## Discussion

The knowledge base [24] defined by RDF does not, *per se*, make distinctions between assertions (ABox) and terminology (TBox) components. In other words, RDF does not by itself differentiate between what is a domain and what is an instantiation of knowledge. This is one of the three reasons why we resorted to S3DB as a mediator [4] in the creation and management of the RDF. The distinction between TBox and ABox triples corresponds, respectively, to S3DB rules and S3DB statements [5,25]. The second reason for resorting to this data service application for the study described here is the convenience of its REST API, which bodes well with the intent to produce an application that is native to the web-browser environment and which therefore can be easily used by a wide variety of domain experts in a number of computational environments. The last reason is that this work is precisely configured to test the hypothesis than an editable schema and streamlined interoperability do make a difference [3].

The main limitation of the study is the restriction of only accepting an RDF that already distinguishes between the ABox and TBox. Furthermore, and with regard to the AGUIA ontology itself, the user cannot assert types of actions beyond the 11 that are listed in Figure 2, even if it is clear that a higher level abstraction would make a more generic use of the browser's document object model (DOM) native methods. The strengths of AGUIA when compared to other applications, such as OntoWebber [15] and WebML [14], include AGUIA's ability to assemble multiple layouts to reflect the multiple contexts of various users. In AGUIA, the user interface is more than a presentation of the page resources, it specifically responds to the semantics behind the information that is represented. As a consequence, when compared to an IIPS study [2], for example, an AGUIA browser-based application is not just more nimble but it is also simpler to use. When using IIPS, the user has a set of tools and needs to know how to use each one to get a satisfactory result in the assembly site. We argue that the literal and semantic attributes of the data can automatically drive that selection.

The AGUIA web application described in this report works with the RDF produced by the S3DB web service. Furthermore, the AGUIA graphic model, composed of combinations of GUI actions and GUI rules (Figure 4), is itself described by the RDF schema (Figure 2). Therefore, the only fundamental barrier to its application in the much wider RDF world in general is the identification of procedures that automatically annotate knowledge bases represented in that format so that they recognize its terminology and assertion components. It could be argued that RDF representations are inherently assertive [26], which poses a fundamental obstacle to its use in the elaboration of terminology. However, it is also clear that the view of the semantic web as a "web of linked data" [27] is particularly conducive to the universal aggregation required by the systems nature of both biological processes and biomedical infrastructures. Our experience with the GI clinical trials initiative described herein and also with earlier work with lung cancer research data [5] is that the accumulation of diverse data sources renders the emergence of comprehensive terminology irresistible.

It is therefore the expectation of the authors that in a domain as fluid as the life sciences in general and molecular biology in particular [3], data processing applications will have to include an iterative tool that will allow domain experts to experiment with the annotation while inspecting a self-assembled graphical user interface. If that direction is to be taken, then the graphic model we have described could be conceived as an integral component of the knowledge base - a GBox [13]. Motik proposed "GBox" to indicate the graphic specification aspect of a knowledge base, as added to the conventional ABox and TBox distinctions. Accordingly, the layout of a GUI web application is conceivably totally describable as a formal GBox component of a knowledge base. The GBox, as shown in Figure 4, was reduced to 11 or fewer components. AGUIA demonstrates how these components alone can determine how the web application was assembled (Figure 6). Such a three-component paradigm would argue that the distinction between terminology and assertion will both have an effect and be a function of the graphic presentation. It would also follow that because different domain experts request different graphic representations (GBoxes) of the knowledge base, they may also be indirectly stating that they place the boundaries between the ABox and TBox differently.

The AGUIA application allows the clinical/domain user's requests and their replies to be automatically translated into graphic interfaces. The main challenge now is to automate as much as possible the processing of RDF "spaghetti" in order to distinguish domain from instantiation such that the automated interface assembly may respond sensibly. We are aware that this is a major challenge since the Web and the content it hosts is assertive (ABox). Accordingly, the main contribution of AGUIA will be to assist in isolating the automatic generation of a TBox that can be interactively checked by domain experts precisely because its automated graphic rendering is in place. The rendering process itself is greatly facilitated by recent improvements in the browser's graphic capabilities, particularly after the introduction of HTML5 and XForms (W3C), and by the open source libraries for user interface assembly that make use of them such as Orbeon [28]. In summary, AGUIA provides a formal, automatable bridge between RDF documents and the browser's DOM-centric extensible syntax.

## Conclusions

This paper describes a web application that automatically assembles user interfaces for databases that are able to generate RDF documents that distinguish between ABox and TBox components. The tools used in this application anticipate the maturation of technologies that either have been recently developed or are still at an incipient stage of development. An example of the former is the strict use of JavaScript to develop the application such that it resides entirely on the web browser. This anticipates a trend toward using server-side components of computational environments as a representation omnibus. An example of the latter is the use of W3C's resource description framework (RDF) as read/write representation media. Currently, the SPARQL query language specifies only the read operation format. In anticipation of the write component being similarly standardized in the future, we have used a research prototype, S3DB, which allows both read and write operations on RDF-like representations.

By developing the autonomously assembled interface applications in response to specific requests from various users who were interacting with a multiplicity of domains and platforms while working with gastrointestinal clinical trials, a number of conclusions became apparent. In regard to the identification of user-friendly, domain-aware interfaces, it appears that it is more effective to develop graphic annotations before settling for a rigid distinction between assertion and terminology, which is in contrast to the more conventional approach to ontology modeling. In regard to the challenge of deploying the applications themselves, it became apparent that modularizing the interface assembly using REST protocols is particularly effective because it does not require a distinction between the universal resource identifiers (URI) that target data elements and those that configure the assembly of the graphical user interface. In conclusion, the long standing artificial intelligence (AI) challenge of contextually aware interfacing appears to benefit from the same RDF-based collaborative annotation that is behind *The Web of Linked Data*. The data-driven user annotation of graphic rules (GBox) was observed to benefit the automation of the graphic interfaces used to interact with those same data elements.

## Abbreviations

AGUIA: Autonomous Graphical User Interface Assembly; RDF: Resource Description Framework; S3DB: Simple Sloppy Semantic Database; HTML: HyperText Markup Language; DHTML: Dynamic HyperText Markup Language; DOM: Document Object Model; CSS: Cascading Style Sheets; PHP: Hypertext PreProcessor; REST: Representational State Transfer; S3QL: Simple Sloppy Semantic Query Language; URI: Universal Resource Identifiers; GUI: Graphical User Interface; TBox: Terminological Component; ABox: Assertion Component; API: Application Programming Interface; GBox: Graphic Component. CTSDS: Clinical Trials Semantic Data Services; W3C: World Wide Web Consortium.

## Competing interests

The authors declare that they have no competing interests.

## Authors' contributions

MCC participated in the design and developed the application, identified the data model, and drafted the manuscript; HFD participated in modeling the database; HFD assisted with the S3DB web service; YA, SVP and JAA provided clinical data and domain expertise; ATV and JSA supervised the project and improved the manuscript. All authors read and approved the final manuscript.

## Pre-publication history

The pre-publication history for this paper can be accessed here:

http://www.biomedcentral.com/1472-6947/10/65/prepub
